# Advancement and properties of circular RNAs in prostate cancer: An emerging and compelling frontier for discovering

**DOI:** 10.7150/ijbs.52266

**Published:** 2021-01-19

**Authors:** Hong Zhou, Xu-dong Zheng, Chang-ming Lin, Jie Min, Shuang Hu, Ying Hu, Liang-yun Li, Jia-si Chen, Yu-min Liu, Hao-dong Li, Xiao-ming Meng, Jun Li, Ya-ru Yang, Tao Xu

**Affiliations:** 1 Department of Pharmacy, Anhui Provincial Cancer Hospital, The First Affiliated Hospital of USTC West District, University of Science and Technology of China, Hefei 230031, China.; 2 Inflammation and Immune Mediated Diseases Laboratory of Anhui Province, Anhui Institute of Innovative Drugs, School of Pharmacy, Anhui Medical University, Hefei, 230032, China.; 3 Institute for Liver Diseases of Anhui Medical University, Hefei 230032, China.; 4 Department of Urology, the Fourth Affiliated Hospital of Anhui Medical University, Hefei, 230011, China.; 5 Department of Urology, The Second Hospital of Anhui Medical University, Hefei 230601, China.; 6 Department of Clinical Trial Research Center, The Second Hospital of Anhui Medical University, Hefei, 230601, China.

**Keywords:** circular RNA, prostate cancer (PC), biomarker, microRNA, tumorigenesis

## Abstract

Prostate cancer (PC) is the most common carcinoma among men worldwide which results in 26% of leading causes of cancer-related death. However, the ideal and effective molecular marker remains elusive. CircRNA, initially observed in plant-infected viruses and Sendai virus in 1979, is generated from pre-mRNA back-splicing and comes in to play by adequate expression. The differential expression in prostate tissues compared with the control reveals the promising capacity in modulating processes including carcinogenesis and metastasis. However, the biological mechanisms of regulatory network in PC needs to systemically concluded. In this review, we enlightened the comprehensive studies on the definite mechanisms of circRNAs affecting tumor progression and metastasis. What's more, we validated the potential clinical application of circRNAs serving as diagnostic and prognostic biomarker. The discussion and analysis in circRNAs will broaden our knowledge of the pathogenesis of PC and further optimize the current therapies against different condition.

## Introduction

Prostate cancer (PC) is the most common carcinoma among men worldwide which results in 26% of leading causes of cancer-related death 1. It is reported that the incidence and mortality of PC is noteworthy higher 2. Accounts for 1.3 million new cases of PC is diagnosed and almost 359,000 associated deaths are responsible for PC development all over the world in 2018 3. Although diagnostic techniques and treatment regimens become increasingly complete and systematic, and a number of patients have achieved a better prognosis and survival rate after androgen stripping therapy, the recurrence, metastasis and castration resistance of PC still bring considerable pressure to patients 4. Therefore, it is urgency to find a novel molecular biomarker for helping to understand the pathological bases of PC and improving the prognosis after comprehensive treatment.

Accumulating data have proved that an intricate interplay among diverse RNA species, including protein-coding messenger RNAs and non-coding RNAs such as long non-coding RNAs (IncRNAs), pseudogenes and circular RNAs, furnishes novel insight in gene regulatory network and significant implication of human disease by acting as competing endogenous RNAs (ceRNAs) or natural microRNA (miRNA) sponge 5. Of note, circRNAs, structurally construct covalent closed loop from 5' to 3'polarity, were originally seen as represent errors in splicing and considered to below abundance. However, informed researches verified that the progression of tumor was in alignment with the amount of circRNAs, which highlighted the regulatory role in carcinogenesis.

**Figure 1:**
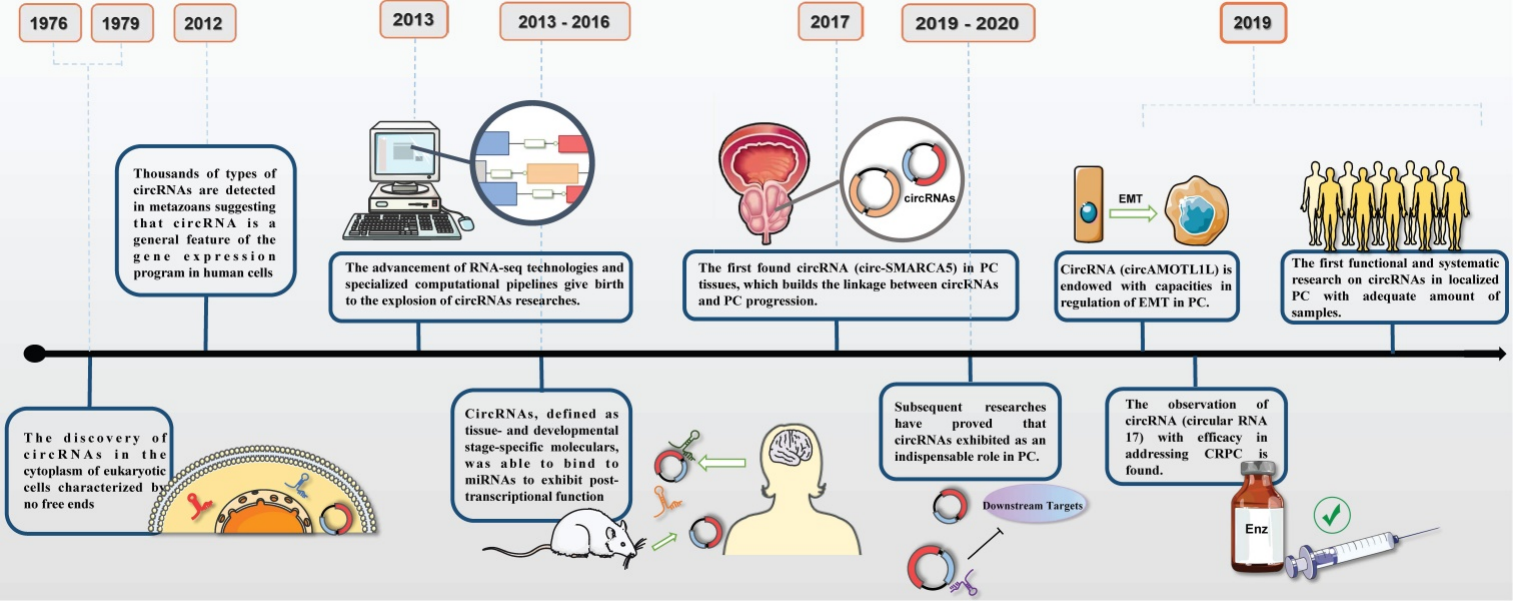
Timeline of circRNAs and its researches on prostate cancer.

The discoveries of circRNAs in PC underwent a tough and twisted period of time. The first observation of circRNA was in 1979 experimented by Hsu and Coca-Prados under electron microscopy 6. Then, early researches on lncRNAs, a particular type of non-coding RNA, elucidated the underlying therapeutic function in progression of PC, which hinted of the same usage in circRNAs. With sufficient efforts on discovering, dysregulation of circRNAs were reported as the leading force in affecting tumor growth, cell proliferation, invasion, migration and apoptosis in PC via modifying the expression of oncogenes andsuppressor genes 7. Most recently, circRNAs have become promising candidates in a vast number of cellular processes and been implicated in epigenetic regulation in PC (**Figure 1**). FOXP4, a member of family of forkhead box transcription factors, was initially reported to be a critical regulator in NSCLC and FOXP4 silencing implied a practical role in carcinogenesis. It had been demonstrated that circABCC4 overexpression was especially common in PC tissues and exerted detrimental function via binding miR-1182, which indirectly inhibited the expression of FOXP4 8. Similar function was also observed in circ0005276 in PC. X-linked inhibitor of apoptosis protein (XIAP), exerted as the critical regulator in promoting tumorigenesis, was the host gene for circ0005276. And further research detected the positive interaction between circ0005276 and XIAP. This intense relevance implied us that circRNAs could be able to directly or indirectly modulate and affect the process of PC 9. Exactly, all these parallel studies were required to testify the hypothesis and set up the regulatory network of circRNAs in PC.

Therefore, in this review, we outline that circRNAs are capable of eliciting a sequence of cellular processes consist of tumorigenesis, metastasis, epithelial-mesenchymal transition (EMT) and castration resistance in PC. Subsequently, we will discuss the potential application of circRNAs as promising targets and biomarkers in clinical strategy. Persistent observations in ascertaining regulatory role of circRNAs help us to figure out the pathogenesis of cancer generation in prostate and provide eventually treatment strategy in clinical application.

## Overview of prostate cancer

PC is the most common non-skin carcinoma among men worldwide, which exerts significant burdens for patients and health-care systems in many countries 10. Globally, PC is the sixth most common cause of cancer-related death 3. Age, race, and family history are currently recognized risk factors for PC. However, a great deal of epidemiologic studies had revealed that the pathogenesis and progress of PC are closely related to alcohol drinking, obesity, prostatitis, benign prostatic hyperplasia (BPH). Digital rectal examination of the prostate (DRE), PSA, prostate biopsy, transrectal ultrasonography (TRUS) and multiparametric magnetic resonance imaging (mpMRI) were the frequently-used diagnostic methods of PC by clinician. However, due to the elevated level in non-neoplastic conditions, including BPH, prostatitis and prostate infarction, the sensitivity and specificity of PSA remained controversial. Besides, beyond the strategies for conservative deferred treatment and radical surgery, the androgen deprivation therapy establishes a golden standard in treating advanced PC. Consequently, it is critical to identify biomarkers with enhanced sensitivity and specificity to diagnose prostate cancer at earlier stages of the disease, and to develop therapeutic tactics that are both safer and more effective than those currently available.

The occurrence of PC follows a multistep process, moving from prostatic intraepithelial neoplasia (PIN) to advance prostate adenocarcinoma with local invasion, and enduringly to culminate in metastatic PC. The Gleason score is the strongest prognostic factor currently, which has shown a positively correlation in disease-free survival 11. To be extent, special consideration has been given to the latest insights regarding the crucial role of inflammation for driving PC. The inflammatory mediators including lymphocytes and macrophages play impeccable role on giving rise to the initiation, induction of aggressive PC phenotype, promotion of tumor metastasis and resistance to chemotherapy of PC. The carcinogenesis of PC may involve in numerous genetic and epigenetic alterations. Many Noncoding RNAs (ncRNAs) are aberrantly expressed, and show evidence of function in oncogenesis or tumor progression. Recent studies have highlighted the prevalence and highly tissue/cell type-specific expression of circRNAs in PC. Specifically, the downregulation or upregulation of circRNAs expression attributes a tumor-suppressor or an oncogenic role to influence the clinicopathological appearance, prognosis and outcome of PC. For instance, circABCC4 could regulate PC progression by modulating FOXP4 signalling pathway 8. CircAMOTL1L significantly decreased the migration and invasion in PC-3 and DU145 cells 12. Therefore, it's worth mentioning that the role of these circRNAs in PC is reviewed in greater detail.

## Overview of Circular RNAs

**Figure 2:**
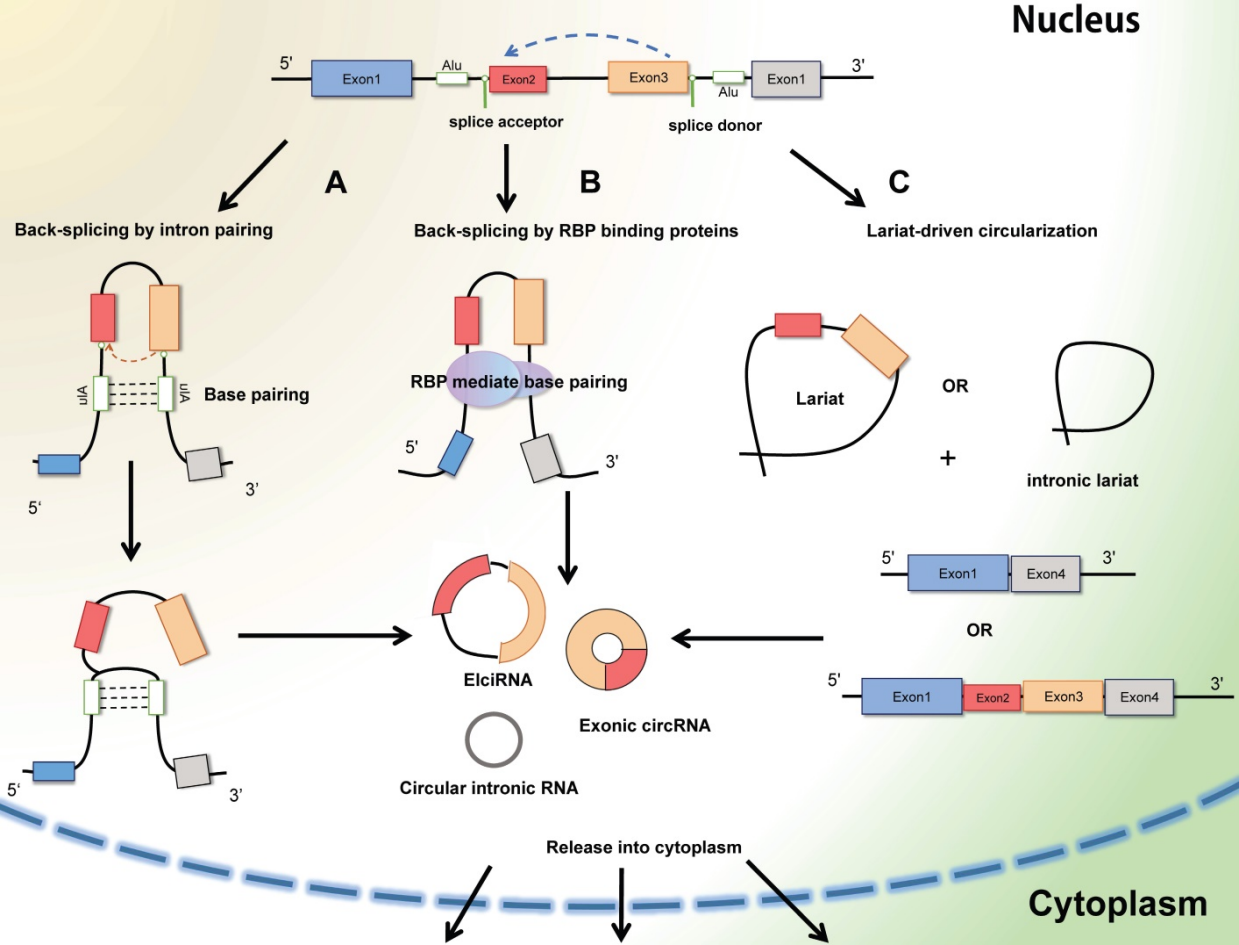
The formation of CircRNAs-Back-splicing and Lariat-driven circularization. A: Conventional and traditional formation of circRNAs model manifests that circRNAs (Exon-intron circRNAs and ExoniccircRNAs) are mainly produced by direct back splicing with the connection of complementary base pairing between inverted repeat elements (Alu elements). B: Besides, trans-acting RNA binding proteins (RBPs) can also generate circRNAs through dimerization between introns during back splicing. C: In some other cases, the mechanism of Lariat-driven circularization model prompts the formation of ExoniccircRNAs and circular intronic RNAs during exon skipping and canonical linear splicing.

Circular RNAs (circRNAs) are generally defined as non-coding RNAs which are endogenous single-stranded circular molecules without either poly-adenylated tails in 3' ends or the cap structure at 5' ends. And it was initially observed in plant-infected viruses and Sendai virus in 1979 by Hsu et al. 6. The circRNAs in host genes accumulate in various types of extracellular body (urine, saliva, and blood) 13, and the amount of circRNA is far more than the linear mRNAs of the corresponding host genes. Considering their multiple localization, circRNA can be segmented into exoniccircRNA (ecircRNA), circular intronic RNA (ciRNA), exon-intron RNA (EIciRNA) and intregenic RNA (icircRNA) (**Figure 2**). In most cases, the lariat-driven circularization model contains direct circularization of intronic lariat and circularization through exon skipping, facilitates the formation of circRNA. The hetero-lariat is formed including both introns and exons (ElcircRNA) in exon skipping. In other condition, direct back-splicing generates the formation of ciRNAs, EcircRNAs and EIcircRNAs 14. Aforementioned features and abundant exist to make circRNAs become promising candidates in diagnosis and prognosis in cancer disease.

CircRNAs have been implicated in multiple cancer processes, including cellular activity activation, EMT regulation and nuclear and cytoplasmic trafficking. Recent studies have indicated that circRNAs can mediate a “sponge” regulatory network (sequestering microRNAs), which can differentially affect the expression of many protein-coding cancer-driving genes and key components of cancer-driving pathways during carcinogenesis. Recent independent reports have identified the circSMARCA5 was a circRNA derived from exons 15 and 16 of the SMARCA5 gene. The downregulation of circSMARCA5 in hepatocellular carcinoma (HCC) is significantly correlated with aggressive characteristics and serve as an independent risk factor for HCC patients after hepatectomy 15. In bladder cancer, circSLC8A1 may act as sponge to regulate the expression level of miR-130b/miR-494 16. Moreover, some circRNAs are linked to reactivation of the androgen receptor signaling axis in PC 17. Considering their dynamic role in cancer, circRNAs may be regarded as therapy target, helping to maintain stable disease and prohibit metastatic spread in tumorigenesis.

## The correlation between Circular RNAS and prostate cancer

With the detection of new technology, the amount of identification of circRNAs in PC is increasing. Because of their abundance, stability and evolutionary conservation, people suggest that they may also have various efficacies in regulating the progression of PC. Besides, it is universally acknowledged that the androgen deprivation therapy ranked to the most prevalent treatment on PC therapeutic measures strongly subjecting to the effect of transformation from androgen dependent to androgen independent. However, with the concomitant increase of statistical correlation between PC and circRNAs, genetic and epigenetic researches are constructed to analyze the potential targets of circRNAs in alleviating the procession of tumorigenesis. Nevertheless, the generally accepted perspective of the correlation with circRNAs and PC is certified, the integrated signaling pathways and molecular mechanism of circRNA-modulated gene regulation are still remaining indistinct (**Table 1**).

## The oncogenic or tumor suppressive roles of Circular RNAs in prostate cancer

Novel study had investigated that circ0005276, located at chromosome X, resided in both cytoplasm and nucleus. According to microarray analysis applied to detect the differential expression of mRNA in PC tissues, mRNA XIAP acquired sufficient attention with the highest fold change. Besides, the uplifted level of XIAP was found in 90 PC tissues and University of California Santa Cruz (UCSC, http://genome.ucsc.edu/) confirmed that XIAP was the host gene of circ0005276. In addition, the relative high expression of circ0005276 was also determined the production of XIAP suggesting that circ0005276 might exhibit oncogenic function in PC progression via enhancing the expression of XIAP. What's more, CCK-8 and EdU assays were carried out to validated that tumor growth was stagnated after silencing circ0005276 or XIAP 9. Furthermore, Feng et al uncovered the tight conjunction between circ0005276 and Fused in sarcoma (FUS) by RIP assay. FUS was firstly known as an RBP contributing to the development of gastric cancer 9. Both of Circ0005276 and FUS protein were identified as up-regulated component in PC tissues. Notably, they detected the effect of transfecting with sh-circ#1 and sh-FUS#1, finding that the levels of XIAP were consistently decreased in either ways. Precisely, circ0005276 and FUS interacted with XIAP mRNA by binding the promoter sequences of it and subsequent researches validated that the luciferase activity of XIAP was attenuated after silencing circ0005276 or FUS. Finally, rescue experiments revealed that over-expressing XIAP reversed the negative efficacy of cell proliferation induced by inhibiting circ0005276 or FUS. Thus, it was indicated that circ0005276 cooperated with FUS exerted oncogenic regulation to modulate the tumorigenesis via XIAP.

CircABCC4 (circBase ID: hsa_circ_0030586; chr13:95813442-95840796), located on chromosome 13q32.1, took crucial roles in PC carcinogenesis. According to Huang's report, the expression of circABCC4 was differently high in both PC and cell lines compared to the control 8. Amusingly, the rate of 5-year in patients showed strong correlation with the high expression of circABCC4 by Kaplan-Meier survival, which was often applied to estimate the probability of survival 19. Besides, Huang et al also revealed that the number of circABCC4-deficient PC3 and DU145 cell lines derived from PC was stagnating at the G0/G1 stage of the cell cycle while few cells are found in the S and G2/M stages, which made the conclusion that the circABCC4 promoted the proliferation of PC cells. After that, they conducted vivo experiment and the result made the same 8. All aforementioned experiments showed that circABCC4 regulated the process of the carcinoma. However, the molecular mechanism of circABCC4 in regulating carcinogenesis still remained undefined.

CircHIPK3 (circRNA ID: hsa_circ_0000284), located at chromosome 11, was firstly found to be of high abundance and have significantly regulatory potency in non-small cell lung cancer (NSCLC) 20, 21. According to previous reports 22-27, the expression level of circHIPK3 was relatively higher in various tumor progression than the control and only circHIPK3 functioned as oncogenic factor by targeting (such as miR-558, miR-124, miR-379, miR-654). A novel research revealed that circHIPK3 could exhibit oncogenic properties by promoting the proliferation, migration, and invasion of PC. Cai et al indicated that remarkable over-expression of circHIPK3 was detected by RT-qPCR in PC tissues and cell lines (PC-3 and DU145) 28. Besides, Chen et al found that the migration and invasion of PC was decreased in circHIPK3 knockdown group by Wound-healing assays and Transwell invasion assays. The result of CCK-8 and colony formation assays indicated the circHIPK3 silencing inhibits the PC proliferation 29. In conclusion, circHIPK3 exerted oncogenic properties in PC cell proliferation and invasion.

Numerous studies had proved that Androgen receptor (AR) signaling pathway played an important role in progression of PC by regulating miRNAs and lncRNAs 30-33. SMARCA5 had the potential capacity of regulating PSA post-transcriptionally in PC progression 34. Due to the GSE18684 dataset, SMARCA5 gene and Quaking (QKI) were androgen-responsive genes. QKI generated the formation of SMARCA5. Circ-SMARCA5, encoded by the gene SMARCA5, was located at chr4:144464662-144465125. In PC tissues, circ-SMARCA5 was overexpressed detected by RT-qPCR and acted as an oncogenic circRNA. Amusingly, with the stimulation of DHT, the expression of circ-SMARCA5 was increasing, which revealed that the circ-SMARCA5 was androgen regulated gene. The conclusion certified the foregoing hypothesis. Additionally, Kong et al. confirmed that circ-SMARCA5 enhanced the proliferation and cell apoptosis in PC. Helping by flow cytometry, they found that knock-down circSMARCA5 increased the percentage of cell cycle in G1 phase and decreased it in S phase. It made the conclusion that circSMARCA5 functioned as oncogenic circRNA in promoting cell cycle progression in PC 35. In short, androgen treatment stimulated the expression of circ-SMARCA5 and the elevated level of circ-SMARCA5 enhanced the cell proliferation and progression in PC.

CircZNF609, located at chromosome15, was initially proved to play a promoting tumor efficacy in breast cancer 36, colorectal cancer 37, renal carcinoma 38 by binding miRNAs. Soon later Jin et al. found that circZNF609 had the same function in PC. CircZNF609 was highly expressed in PC tissues and si-circZNF609 suppressed cell viability and colony formation and enhanced apoptosis in PC cell lines (PC-3 and LNCaP) by Western blot. They also found that the expression level of MMP-9 and Vimentin decreased after silencing circ-ZNF609 39. MMP-9 degraded the collagen of basement membrane, which destruction was usually an essential step on supporting tumor invasion and metastases 40. Emerging evidences have shown that MMP-9 played a role in tumor invasion, metastasis and angiogenesis and mediated tumor microenvironment in basal-like triple negative breast cancer, Lewis lung carcinoma etc. 41-46. It indicated that si-circZNF609 restrained cell migration and invasion in PC 39. Taken together, up-regulated circZNF609 enhanced proliferation, migration, invasion and apoptosis of PC cells.

Hsa_circ_0001206, located at chromosome 22, expressed a low level of PC cell lines compared with the control. According to Song's report, they utilized hsa_circ_0001206-overexpression lentivirus to validate its dramatical down-regulated level in PC tissues and cell lines (PC-3, DU145 and LNCaP) by qRT-PCR. Moreover, Song et al revealed that the migration ability and cell proliferation ability was weaker than NC cells in over-expression hsa_circ_0001206 cells 47. To discover its mechanism of down-regulated level of hsa_circ_0001206, they focused on DHX9. DHX9, a member of DExD/H-box helicase family, appeared to play a significant role in many biological processes including regulation of DNA replication, transcription, translation, microRNA biogenesis, RNA processing and transport, and maintenance of genomic stability 48. Previous studies have shown that DHX9 is positively correlated with the development of colorectal cancer, hepatocellular carcinoma, Ewing sarcoma and PC 47, 49-51. Song et al used DHX9-siRNAs to knock down DHX9 and detected its efficiency by qRT-PCT in DU145 cells. A marked reduction in hsa_circ_0001206 was observed when transfected cells with DHX9-siRNA2 compared with siRNA-negative control 47. In short, hsa_circ_0001206 functioned as suppressive ability in PC cell proliferation, migration and invasion modulated by DHX9.

CircAMOTL1L, located at chromosome 22:35948707-35948901, was produced by exon 3 of angiomotin-like 1 gene (Amotl1) and mainly existed in cytoplasm. As initial studies had revealed, circAMOTL1 was seen as protective factor in human cardiac tissues by reducing apoptosis induced by doxorubicin (Dox) and enhancing cardiac repair through binding to and activating AKT phosphorylation and nuclear localization 52. Inspired by aforementioned experiments, Yang et al. discovered circAMOTL1L circularized from exon-2 and exon-3 of gene Amotl1 53. They speculated that circAMOTL1L might be involved in migration of PC based on previous study shown that AMOTL1 was important in cadherin-11-meidated cell migration 54. Therefore, they confirmed it through constructing recombinant plasmid and fortunately they found that circAMOTL1L downregulated in prostate tissues and functioned as suppressor in carcinogenesis and progression. Additionally, they revealed that p53 modulated the expression of RBM25 to activate circAMOTL1L biogenesis found by transfecting siRNA and using lentiviral vector system in PC-3 cells 12. P53 played momentous role in development and progression in various cancers due to its abilities of losing and mutating 55, 56. And novel researches demonstrated that RBM25 participated in large fraction of alternatively spliced exons throughout the human genome by interacting with the exonic splicing enhancer, CGGGCA sequence, which located within exon 57, 58. In sum, circAMOTL1L mediated by p53-RBM25 postponed the PC progression.

Circ-102044, located at chromosome17, was highly up-regulated in PC. A novel study had demonstrated that circ-102044 functioned as oncogene in promoting migration and invasion in progression of PC and joins in a head-to-tail manner at the slice junction 59. Besides, western blotting also revealed that the up-regulation of BCL2 and MMP2 were observed in both cell lines. The high expression level of MMP2 was positively related to metastasis of PC and BCL-2 was a protective factor that prevented PC from apoptosis through the combination of molecules and signaling pathways, such as in PTEN loss and p53 inactivation, PI3K/AKT phosphorylation, activation of RTK/STAT3/NF-kB, Ras/Raf1/MEK/ERK, microRNAs (miR-24, miR-31, miR-34, miR-195, miR-204, miR-205 and lncRNA MEG3), autophagy proteins (Beclin1 and AMBRA1) and other potent molecules (FKBP38/NR4A1/GAL3) 60, 61. But the molecular details of pathways by which circ-102044 modulates PC still remain unknown.

CircRNA-MYLK (circRNA ID: hsa_circ_0141940), located at chromosome: 123332641-123332832, was significantly up-regulated in PC tissues and cell lines (DU145, LNCaP, PC-3 and PC-3MIE8) compared with the control (WPMY-1 cells) by RT-qPCR. To deeply clarify its potential functionsand regulatory mechanisms, Dai et al conducted functional experiments and revealed that circRNA-MYLK overexpression improved cells proliferation and colony formation, whereas circRNA-MYLK acted as suppressor in cell apoptosis 62. Furthermore, the invasion and migration abilities of overexpressing-circRNA-MYLK were significantly stronger than the control by detecting the invasive and migratory number of PC cells (PC-3MIE8), which were reversed after silencing circRNA-MYLK. In sum, circRNA-MYLK exhibited as oncogene in prompting PC progression, migration and invasion.

Researchers identified a novel circRNA, named circMBOAT2, which was increased significantly in PC tissues and PC cell lines (VCaP, LNCaP, C4-2B, DU145 and PC-3) 63. When circMBOAT2 was silenced, the ability of proliferation in PC-3 and DU145 cells was inhibited. Meanwhile, migratory and invasive capability of PC-3 and DU145 cells were significantly attenuated. To explore the effect of circMBOAT2 *in vivo*, luciferase-labeled PC-3 cells transfected with short hairpin circMBOAT2 (sh-circMBOAT2) were subcutaneously injected into BALB/c nude mice. Unsurprisingly, the volume and weight of tumors were notably decreased in the sh-circMBOAT2 group. And thus, circMBOAT2 may impair proliferation of PC cells and act as an oncogenic gene. In addition, Jinet aldiscovered that circLMTK2 was significantly downregulated in PC patients' tissues. Further results confirmed that circLMTK2 overexpression could suppress proliferation in LNCaP and PC-3 cells, while markedly elevate the apoptosis-related protein levels, including Bax, Cleaved-caspase-9 and Cleaved-caspase-3. Furthermore, Western blotting assay results showed that the cell migration and invasion in LNCaP and PC-3 cells were remarkably inhibited when the expression level of circLMTK2 was increased. Increasing evidences showed that miRNA could bind to the sites of circRNAs. MiR-183 is a well-conserved microRNA across many species from invertebrates to humans. Many studies have unveiled the functions of miR-183 in different types of tumors, such as colorectal cancer (CRC) 65, non-small cell lung cancer (NSCLC) 66, osteosarcoma 67, and so on. MiR-183 involved in the progression of LNCaP and PC-3 cells, and might be reduced by increasing the expression level of circLMTK2. Taken together, circRNAs could be regarded as oncogene or suppressor gene in tumorigenesis.

As stated, circRNAs are appropriate to be used as oncogenic stimuli or suppressive factor in modulating PC progress, which may be hinted as exhibiting possible biomarker and therapeutic targets in the future.

## The epigenetical roles of Circular RNAs in prostate cancer

CircRNAs extensively participate in gene expression process among numerous animal species with gene-specific and cell-type specific manner 68. With the increasingly functional researches launched, the epigenetic function of circRNAs is under investigation. It is ubiquitous that circRNAs have numerous miRNA binding sites which can be attached to miRNAs like a sponge due to the observation of structure 69 (**Table 2**). Helped by the construction of absorbing miRNAs, downstream targets are activated or suppressed to regulate the progression of PC (**Table 3**). In addition to that, the epigenetic features of circRNAs contains regulation of selective splicing or transcription; acting as Sponges for RBPs; protein translation modification; circRNA-derived pseudogenes; involving in the process of exosome function 70.

It had been identified that miRNAs functioned as oncogenes or suppressors in human cancers 71. MiR-1182 was initially been regarded as tumor suppressor in gastric cancer proliferation by negatively modulating hTERT 72. CircABCC4, also known as hsa_circ_0030586, was highly up-regulated in PC tissues according to a public PC database (GSE77661). Firstly, miR-1182 was significantly down-regulated in PC tissues, whereas circABCC4 was obviously up-regulated. Furthermore, Luciferase assays demonstrated that mutation of the AGACCUC motify impeded the connection between circABCC4 and miR-1182. The continuous knockdown results favored the view that the deficiency of circABCC4 caused a increase in miR-1182 expression leading to inhibit the cell proliferation, migration and invasion of PC. Then, Luciferase experiments provided the evidence that the FOXP4 expression exhibited the negative correlation with miR-1182 8. FOXP4, a forkhead transcription factor, has been demonstrated to play significant roles in embryonic development, cell cycle regulation, and oncogenesis and it is fully expressed in heart, brain, lung, liver, kidney, and testis 73. Finally, Huang et al reported that silencing circABCC4 could dramatically reduce the expression of FOXP4 and overexpression of FOXP4 reversed the inhibitory effect of miR-1182 on FOXP4 expression in miR-1182-overexpressing PC cells (PC-3 and DU145). Functional assays were also conducted to elucidate that overexpression of FOXP4 partly rescued the proliferation, migration and invasion defect in circABCC4-deficient cells. They also found that circABCC4 played an important part in tumor growth by regulating miR-1182/FOXP4 signaling pathway 8. Conclusion, circABCC4 accelerated the pace of progression of PC by down-regulating the expression of miR-1182 and up-regulating the expression of FOXP4.

Previous studies had demonstrated that extracellular vehicles (EVs) embraced several biological molecular such as non-coding RNAs mediated the interaction between cancer cells and their microenvironment 74, 75. To validate the internal mechanism of EVs in PC, numerous exosomes were extracted from patients and its healthy control, finding that 42 downregulated and 69 upregulated circRNAs in the heat map and circ-0044516 exhibited the most promising candidate for lucubrating the post-transcriptional activity with the significantly increased expression in prostate tissues. In addition, the result of colony formation assay showed that silencing circ-0044516 caused a severe reduction in colony numbers in PC cells (PC-3, 2B4 and 22RV1). The similar trend was observed in Transwell chamber with or without Matriger treatment suggesting that inhibiting circ-0044516 had negative effect on PC cell metastasis. What's more, Luciferase reporter assay revealed that circ-0044516 harbored miR-29a-3p to act as a sponge in PC. Interestingly, further study detected an inversely result that serum samples from PC patients also verified the mutually-exclusive relationship between circ-0044516 and miR-29a-3p 76. According to this observation, circ-0044516 was proposed to process predictable function as biomarker in clinical management.

Through the bioinformatics prediction and function analysis, Cai et al suggested that circHIPK3 had the binding site with miR-338-3p. To identify the molecular mechanism of the interaction, they detected the luciferase activity and RT-qPCR data to confirm that circRNA had a negative correlation with the miR-338-3p in PC cells (PC-3 and DU145). Additionally, they indicated that ADAM17, an essential role of the ADAMs family 77, was the targeting gene of miR-338-3p. ADAM17 was becoming a ubiquitous component with tumor cell specificity, which extensively existed in malignant carcinoma 78. Interestingly, the suppressed expression of ADAM17 by knock-down circHIPK3 is reversed by transfection of miRNA-338-inhibitor. What's more, the result of CCK-8 assay demonstrated that the proliferative and invasive capacities of circHIPK3-deficient cell lines (PC-3 and DU-145) were reversed by co-transfecting si-circHIPK3 and miRNA-338-3p inhibitor, which led to the conclusion that circHIPK3 regulated the proliferative and invasive potentials of PC through miRNA-338-3p/ADAM17 axis 28. After that, Chen et al. 29 confirmed the hypothesis that circHIK3 also exerted its role in tumorigenesis by sponging miR-193a-3p. Ensuing Luciferase reports validated that silencing circHIPK3 suppressed the expression of MCL1 mRNA and protein while inhibiting miR-193a-3p elevated it. Furthermore, the results of CCK-8, Wound-healing and Transwell assays indicated that MCL1 overexpression increased the proliferation, migration and invasion of PC cell lines (PC3 and DU145), which was targeted by miR-193a-3p. Myeloid cell leukemia 1 (MCL1), an anti-apoptotic member of the BCL-2 protein family, suppressed intrinsic apoptosis or programmed cell death in previous study. Therefore, they identified that circHIPK3 functioned as miR-193a-3p sponge which regulated the expression of MCL1 oncogene.

Recent studies had shown that miR-186-5p participated in various occurrence of PC 79, 80. Jin et al also found that miR-186-5p presented the protective efficacy in PC and it was modulated by circ-ZNF609. In addition, transfecting miR-186-5p inhibitor reversed the reduction in cell colony and viability and rise in cell apoptosis induced by circZNF609 while western blot favored the consistent result, which meant si-circZNF609 restrained the cell migration and invasion by up-regulating miR-186-5p. What's more, silencing circZNF609 caused relevant reductions in the level of YAP1, whereas transfecting miR-186-5p enhanced it. Then, the similar result was obtained in AMPK signaling pathway. The YAP1 and AMPK signaling pathway activated in PC and had strong association with pathogenesis 81, 82, which furnished fresh thought to Jinet al. Fortunately, they had explored that the ratio of p/t-AMPK and level of YAP1 was declined when sh-circZNF609 involved and the result was reversed with the effect of transfection of miR-186-5p 46. Taken together, YAP1 and AMPK-catenin signaling pathways improved carcinogenesis through miR-186-5p regulated by circZNF609.

Hsa_circ_0001206 had been proved its function in carcinogenesis. To explore its potential interaction with miRNAs, miR-1285-5p was predicted by home-made microRNA target prediction software from Array star and sooner it was confirmed by dual-luciferase. MiR-1285-5p was systematically defined as regulatory factor in tumor progression 83. Previous reports had indicated that miR-1285-5p could influence the overall survival in breast cancer and infiltrative growth of follicular varients of papillary thyroid carcinomas 84, 85. In addition, Smad4 was identified as a potential target of miR-1285-5p by TargetScan database. To sustain the assumption, Song et al. constructed co-transfection model with miR-1285-5p mimic to observe whether the alteration of Smad4 mRNA occurred compared to the initial state in hsa_circ_0001206 overexpressing cells. Fortunately, the level of Smad4 mRNA expression elevated in overexpression of hsa_circ_0001206 cells while transfecting miR-1285-5p declined it 47. Emerging evidences had elucidated that Smad4 exerted a suppressive effect on the progression and metastasis of PC 86. Therefore, these results suggested that hsa_circ_0001206 regulated the tumor growth by competitively binding miR-1285-5p through modulating Smad4 expression. Noteworthily, according to the result of GEPIA web ted, lower Smad4 levels predicted better disease-free survival, which gave focus on the prognostic role of Smad4 or hsa_circ_0001206 as biomarker 47. In summary, combined with aforementioned study, hsa_circ_0001206 intervened with the PC cell proliferation, migration and invasion by interacting with miR-1285-5p, which was negatively regulated by DHX9 protein.

In Yang's report, they searched for the correlation between miRNAs and circAMOTL1L via bioinformatics analysis and they discovered that circAMOTL1L has miR-193a-5p binding sites 12. MiR-193a-5p was upregulated in PC tissues and PC cell lines, with significant suppression of PC3 cell apoptosis induced by oxidative stress. Then, Luciferase assays and RNA *in situ* hybridization presented the presence of combination between circAMOTL1L and miR-193a-5p. Additionally, RBPs (RNA-binding proteins) played significant role in forming circRNAs because of the non-canonical form of alternative splicing 57, 58. Specially, Yang et al discovered that RBM25, one type of RBPs, directly bound to poly-G sequences or the exon splicing enhancer 5'-CGGGCA-3' motif of circAMOTL1L. Moreover, they had validated that miR-193-5p might target Pcdha 3'-UTR, which contained highly conversed miR-193-5p binding sites, to regulate the progression of PC 12. Previous reports had manifested that Pcdha gene cluster functioned as tumor suppressor in tumor growth and metastasis 87-90. In short, it was confirmed that the circAMOTL1L-miR-193a-5p axis suppressed the PC progression by up-regulation Pcdha.

Additionally, based on the miRBase prediction, a potential linkage between miR-29a and circRNA-MYLK on a complementary sequence was discovered and sooner confirmed by transfecting miR-29a mimics 62. High level of miR-29a was initially reported as suppressor in PC progression 91. Besides, the expression of miR-29a was markedly decreased in PC cells transfected with circ-MYLK overexpression vector while si-circRNA-MYLK presented opposite function in PC cells 62. In conclusion, circRNA-MYLK promoted PC proliferation, colony formation, invasion, and migration by downregulating the expression level of miR-29a.

To obtain underlying conjunction between circRNA and miRNA, scientists attempted to find a snap path focused on miR-145. MiR-145, initially seen as participator in improving proliferation and invasion in both PC LNCaP cells and IncRNAPCGEMI, were proposed as potential cooperator with circRNAs 92. He et al conducted microarray analysis to demonstrate five puissant candidates including hsa-circRNA-101981, hsa-circRNA-101996, hsa-circRNA-091420, hsa-circRNA-008068 and hsa-circRNA-406557. Moreover, the results of RT-PCR had validated the downregulation of hsa-circRNA-101981, hsa-circRNA-008068, hsa-circRNA-406557 and upregulation of hsa-circRNA-091420, hsa-circRNA-101996. Besides, hsa-circRNA-101981, hsa-circRNA-101996, hsa-circRNA-091420 were the three circRNAs bound to hsa-miR-145-5p and the others concentrated to hsa-miR-145-3p were all confirmed by dual-luciferase reporter assays 93.

Amusingly, both miR-145-5p and miR-145-3p were defined as anti-cancer molecules to regulate downstream proteins (MELK, NCAPG, BUB1 and CDK1) in PC 94. Inspired by the thoughts of He et al., scientists paid much attention on the regulatory effect of miR-145-3p that was proved to be capable of suppressive role in PC. Due to the result of sequencing, a compact and complementary connection between circ_KATNAL1 and miR-145-3p was found but either of them was significantly depleted in PC cells (PC-3 and LNCaP cells). So, it was deduced that there was synergistic anti-cancer property in molecular combination of circ_KATNAL1 and miR-145-3p in PC which was further confirmed by the results of Pull-down experiments. In addition, the function of transfecting with miR-145-3p and overexpressing circ_KATNAL1 impeded the expression of WISP1 that was a key factor regulating in several types of cancers, such as hepatocellular carcinoma, breast cancer, lung cancer and colon cancer 95. So, it was manifested that circ_KATNAL1 exerted suppressive role in PC via miR-145-3p/WISP1 pathway. What's more, declined activities of MMP-2, MMP-9, Caspase-3, Caspase-8 and Caspase-9 were observed when transfecting circ_KATNAL1 or miR-145-3p, which provided the evidence that these apoptosis-related pathways and invasive enzymes could be activated by circ_KATNAL1/miR-145-3p/WISP1 pathway 96. Taken together, circ_KATNAL1 functioned as suppressor in PC progression by absorbing miR-145-3p to target downstream proteins.

**Figure 3:**
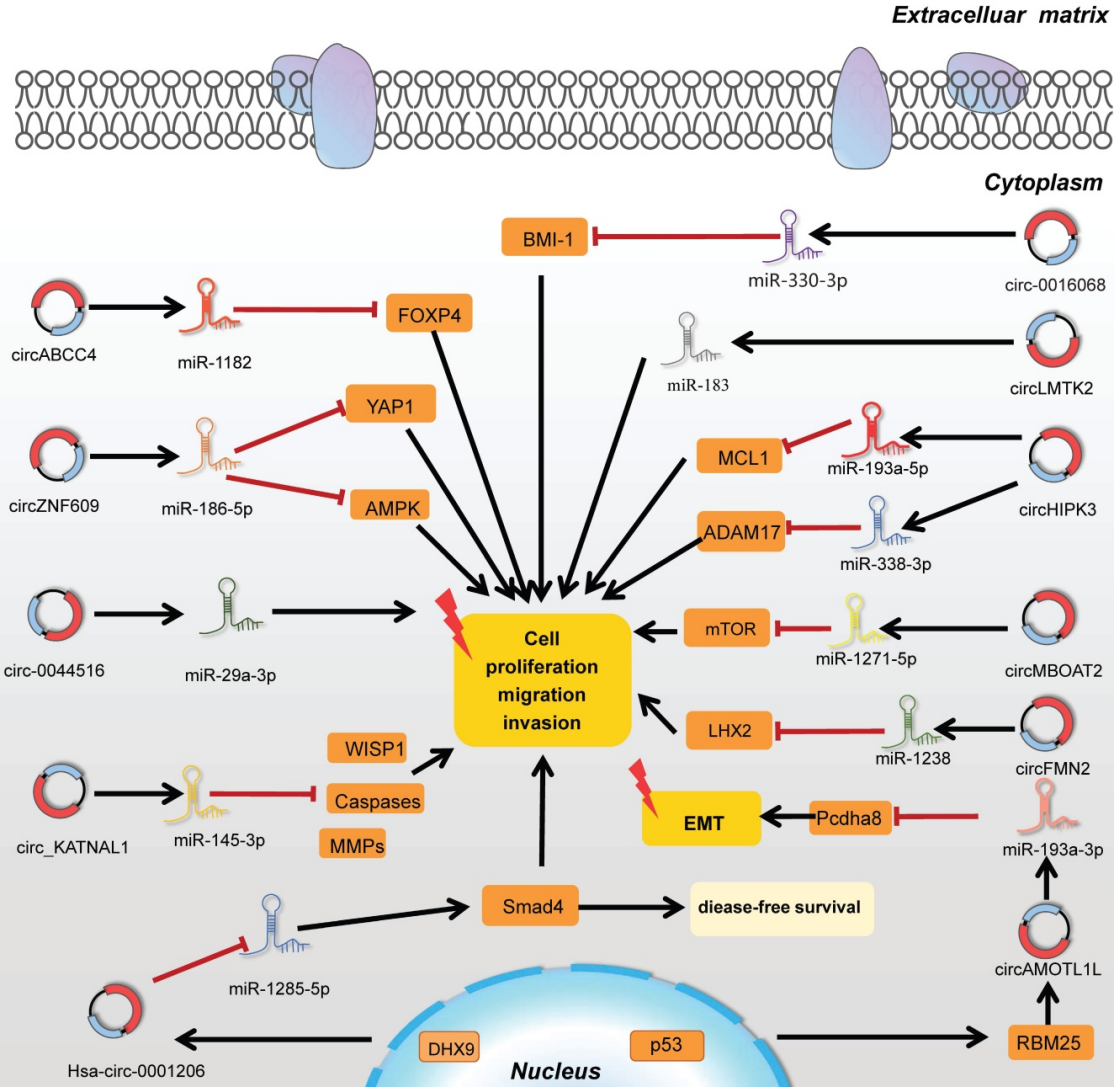
CircRNAs function as miRNA sponges by targeting related genes in tumorigenesis.

Taken together, the studies on circRNA-miRNA-mRNA pattern were indeed extraordinarily displayed in epigenetic regulations, revealing the abundant intramural network of tumorigenesis and providing a wide range of novel clues for the progression of PC. CircRNAs exerted as miRNA sponges in PC were vividly listed in **Figure 3.**

## Roles of Circular RNAs in epithelial-mesenchymal transition

Epithelial-mesenchymal initially transition is defined as a complex molecular process required during the embryonic development for morphogenetic changes 97. To achieve this transition, epithelial, stromal settings and microenvironment molecules that able to accommodate cellular and tissue growth in normal or altered conditions are required 98. Several molecular factors participate in this process such as Twist, Snail, Slug and Zeb1/2. Additionally, numerous studies have demonstrated the presence of EMT-like states in PC, suggesting its involvement in PC development and metastasis 98, 99. Besides, EMT present obvious efficacy in changing from primary PC to invasive advanced cancer. Moreover, Due to the modification of abnormal miRNAs levels can make resistant PC cells more sensitive to small molecule drugs, the combination of these innovative agents (such as small molecules and miRNAs) may represent a favorable approach for the therapeutic treatment of resistant PC.

AMOTL1L gene was initially reported as a partner of N-cadherin complex which contained tight junctions and it involved in controlling paracellular permeability and cell polarity 53, 54. Previous studies had revealed that AMOTL1L gene might be closely related to PC progression 12. And according to Yang's report, circAMOTL1L, expressed by gene AMOTL1L, could act as miR-193a- 5p sponge and modified the biogenesis of Pcdha expression. Furthermore, knock-down of miR-193a-5p could partly rescue the increased expression levels of mesnchymal cells (vimentin) and the decreased expression levels of epithelial cells (E-cadherin). Additionally, the transformation effect would be further enhanced with the addition of circAMOTL1L overexpression. E-cadherin was responsible for adherens junction and functioned as extracellular phenotype maintainer. Vimentin and β-catenin contributed to cellular migration and were seen as mesenchymal markers 100. Down-regulation of E-cadherin and up-regulation of vimentin and β-catenin played momentous role in EMT, which led to reduction of intercellular adhesion and increased in cell migration. So, it was proposed that miR-193a-5p promotes the PC progression, migration and invasion through the induction of EMT 101. Combined with aforementioned researches in yang's report, p53-RBM25 down-regulation attenuated the expression of clustered Pcdha gene to accelerate the PC progression. To figure out whether p53 exerted indispensable role in driving EMT, a rescued experiment was performed via over-expressing circAMOTL1L in p53-depleted PC3 cells and fortunately subsequent researches revealed that the decreased level of E-cadherin and increased level of vimentin and β-catenin were reversed after amplifying the expression of circAMTOL1L. Amusingly, the function of over-expression of circAMOTL1L was opposed to RBM25 knock-down and overexpression of circAMOTL1L further enhanced the expression level of RBM25 protein, which suggested that RBM25 and circAMOTL1L formed a feedback loop to regulate the EMT-related gene expression in PC cells 101.

circFoxo3, located at chromosome16, was initially seen as a suppressive regulator in breast cancer by promoting its parent gene Foxo3's expression to inhibit the level of MDM2 induced by Foxo3 ubiquitination. Recently, Shen et al elucidated the consistent result by establishing circFoxo3 siRNA transfected PC cells. Besides, immunoblotting indicated that an increased level of Foxo3 and E-cadherin and a reduction of N-cadherin and vimentin levels were observed in DU-145 cells transfected with circFoxo3. On the contrary, the appearance opposite to result mentioned above was gained when they silenced circFoxo3 and Foxo3 with siRNAs 102. Taken together, all these data firmly confirmed that circFoxo3 exerted as androgen responsive circular RNA repressed PC viability by enhancing Foxo3 and inhibiting EMT. Surprisingly, regardless of its function in EMT, scientists also revealed that the delivery of circFoxo3 prompted the apoptosis of tumor tissues of docetaxel (DOX) injection group mice and this opposite effect was prevented by injecting circFoxo3 siRNAs, which suggesting that circFoxo3 might be correlated to PC androgen independence and provided an applicable strategy for using Dox associated medicine.

CircFMN2, located at chromosome 1, was primordially discovered in CRC and PC 103, 104. The high expression level of circFMN2 in CRC was relevant to advanced tumor stage and tumor node metastasis (TNM) stage. Subsequently, Shan and his colleagues found that circFMN2 was upregulated in PC tissues. The expression level of circFMN2 in PC cell lines (PC-3, LNCaP, VCaP and DU145) was higher than that in the human normal prostate epithelial cell line (RWPE-1). The results showed that circFMN2 knockdown was remarkably increased the expression level of E-cadherin and greatly decreased the expression level of N-cadherin and Vimentin in PC-3 and DU145 cells. Correspondingly, the expression level of E-cadherin was decreased, and the expression level of N-cadherin and Vimentin were increased in VCaP cell when the expression level of circFMN2 was upregulated. CircFMN2 could play an oncogenic role in the pathogenesis and progression of PC. In addition, the expression level of circ-0016068 was significantly elevated in PC cell lines (DU 145, 22RV1, PC-3, and VCaP) in a separate study 103, 104. The results discovered that the growth of 22RV1 and DU 145 cells were simultaneously inhibited when circ-0016068 was downregulated. Furthermore, circ-0016068 silence stimulated the expression level of E-cadherin and diminished the expression level of Vimentin and Snail, while circ-0016068 overexpression generated the opposite effect in 22RV1 and DU 145 cells. Moreover, nude mouse tumorigenicity assay were used to verify roles of circ-0016068 *in vivo*. The results showed that circ-0016068 could accelerate the expression level of Vimentin and Snail whereas suppress the expression level of E-cadherin. Therefore, circ-0016068 could serve as an oncogene regulator in the course of PC.

It was commonly considered that several signaling pathways were involved in the process of EMT including PI3K-Akt signaling pathway, TGF-β signaling pathway and MAPK signaling pathway. These cascade activations became the backbone of subsequent event of alteration in cytoskeleton, contributing to the changes in polarity, increases in the mobility, and invasiveness of tumor cells, which was called EMT. Recently, Yan et al. 106 revealed the high quantities of hsa_circ_0001085, hsa_circ_0004916 and hsa_circ_0001165 by utilizing high-throughput sequencing techniques. Amusingly, circRNAs mentioned above were capable of the change in the level of essential proteins associated with EMT. Hsa_circ_0001165 was able to absorb miR-187-3p to regulate the expression of TNF. What's more, hsa_circ_0001085 was shown to be an effective molecular in the process of PI3K-Akt signaling pathway by indirectly regulating the AKT1 and PIK3CG expression level. And the similar appearance was observed in MAPK signaling pathway and TGF-β signaling pathway via binding miR-451a and miR-196b-5p to modifying the expression of MAPK1 and TGFBR2 proteins. What was noticeable that IFN-γ firstly referred in U.G.Lo's report was performed to induce EMT in PC during this research, which provided novel avenues for the mechanism researches in the future 107.

## Roles of Circular RNAs in development of castration-resistant prostate cancer

To completely cure or delay the disease, people have developed the treatments of PC including surveillance, radical local treatment, and androgen-deprivation therapy (ADT). Notably, medical (LHRH agonist) therapy and surgical castration both had efficient efficacy in advanced PC. Despite ADT therapy brought survival light to patients with advanced PC, ADT failed to complete their wishes result from its limited function only for 2-3 years and consequently led to castration-resistant PC (CRPC) 108. Therefore, it furnished fresh thoughts to public on how to treat advanced PC avoiding castration-resistant.

Emerging studies had revealed that alternative androgen receptor (AR) and its splicing variants presented critical role in development of CRPC because of their general lack of the androgen-binding domain 109-111. ARv7 had a conspicuous function among other 15 AR-Vs in progression of PC 112-114. To be extent, shorter survival ability and biochemical recurrence were found in PC patients with high expression of ARv7, which indicated that elevated level of ARv7 was remarkably correlated with the stage of CRPC 115, 116. So Wu et al. discovered the importance of predicting the expression level of ARv7 after radical prostatectomy due to its efficacy on developing Enzalutamide resistant prostate cancer (EnzRPCa). Furthermore, in Wu's report, circRNA 17, located at chromosome4, was negatively related to the expression of ARv7. They confirmed that knock-down circRNA 17 enhanced the C4-2 parental cells' resistance to Enz treatment and cell invasion through increasing the expression of ARv7 by Matrigel-chamber assay30674872. Besides, the result of Pull-down assay validated that circRNA 17 directly functioned as a reservoir or stabilized the statue of miR-181c-5p prevented from the degradation from nucleases such as Tudor-SN endonuclease to regulate the combination of miR-181C-5p with 3'UTR of ARv7, which indirectly alters the Enz-resistant and cell invasion30674872. MiR-181c-5p played momentous role in cell proliferation, cell cycle and cancer progression in cancer stem cell formation. Moreover, Transwell and 3D invasion assay indicated that Enz altered the expression of PDLIM5 (host gene of circRNA 17) through circRNA17/miR-181c-5p/ARv7 pathway to modulate the level of ARV7, which eventually resulted in CRPC cell invasion and Enz resistance30674872. And the similar result was obtained *in vivo* experiment by using vivo imaging system, used to monitor the PC progression23792449, for proceeding noninvasive monitoring. Taken together, circRNA17/miR-181c-5p/ARV7 axis presented a novel target that might prevent PC from being castration-resistant cancer.

CircUCK2, located at chr1:165859440 -165877108, was identified as candidate in uncovering secrets and addressing issues on castration resistant effect after long-term anti-androgen therapy in PC patients. According to Xiang's report 118, circUCK2 was shown to be elevated in EnzR-C4-2 cells and was conformed to be related to the invasion and metastasis of PC. Besides, the result of Pull-down assay concentrated the tight connection between circUCK2 and miR-767-5p and revealed that miR-767-5p presented the opposite effect to circUCK2 on cell proliferation and cell invasion by using biotinylated oligo to display results in PC. Initially reports had validated that decreased TET activity was correlated with the progression cancer, which could be induced by activation of miR-767 to repress TET1/3 mRNA, protein expression and to regulate genomic 5hmC levels 119. Moreover, due to the literature and bioinformatics tools (miRbase, Target-scan and RNA22) provided online, TET1 was selected to be further investigated and subsequently scientists detected that the expression level of TET1 was elevated after overexpressing circUCK2 but reversed overexpressing miR-767-5p. Worthily, the decreased level of TET1 accelerated the progression of PC, which brought brand new insight for future clinical treatment by utilizing TET1 agonists or other specific biological products to delay the process of CRPC.

## Roles of Circular RNAs as diagnostic markers in prostate cancer

PC is the most common non-skin carcinoma among men worldwide affecting patients and health-care systems in many countries with low relevant ratio and poor tolerance. Managements consists of digital rectal examination (DRE), PSA measurement, imaging in the form of transrectal ultrasound-guided scan (TRUS) and multiparametric magnetic resonance imaging scan (mpMRI) were deputed to screen and diagnose the potential cases with systematic conjunction 120. Although the quantification of PSA retained the indispensable function in clinical application, it was unable to differentiate between aggressive PC and indolent disease and further associated with a high risk of over-diagnosis and overtreatment 121, 122. What's more, sustaining debates on prostate biopsy rose from the considerations among public leading to concentrate on its controversial patient tolerance and morbidity rather than its golden standard for detection. Besides, combined with other technologies, the Gleason grade group, proposed by International Society of Urological Pathology (ISUP) in 2014, remained the backbone of clinical outcome and treatment response and the clinical features including the Gleason score became the imperative characteristics as idea and competent biomarker 123. Owing to their role of assessing the risk of biochemical progression post curative therapy, the Gleason grade group method prevented unnecessary treatment and informed patients of their actual risk level to evaluate prognostic risk. Hitherto, there was still no reliable molecular for predicting the outcome of PC patients to distinguish patients who need definite treatment from patients who have latent disease 124. And it was indubitable that determining clinical stages contributed to better overall survival and longer disease-free survival then subsequently led a reduction in morbidity among PC patients. Moreover, the current prognostic system still needed to complete on account of immature prognostic precision 125. Recently, circRNAs correlated with cancer pathogenesis had become a compelling field for seeking ideal diagnostic and prognostic markers 126. The characteristics of tissue specificity, stability and evolutionary conservation rose from the extensive concerns in scientific researches resulting in a new dimension to clinical strategies.

Current reports investigated that circ-ITCH, mapped to chromosome20, were differently expressed in PC tissues compared with adjacent noncancerous prostate tissues 127. The level of circ-ITCH showed great efficacy on distinguishing cancer from paired adjacent tissues through ROC curve with the area under curve (AUC) being 0.812. The sensitivity and specificity reach their peak values of 88.3% and 61.7% respectively. Thus, all of these facts drew a conclusion that circ-ITCH could be used as remarkable biomarker to conform to contemporary technical improvement and result in effective screening. To explore the potential mechanism of its relation with clinicopathological development, the expression of circ-ITCH was divided into low expression (N=circ-ITCH<0.764, N=162) and high expression (N=circ-ITCH>0.764, N=162) groups. In addition, Kaplan-Meier curve analysis and log-rank test were conducted to elucidate that high expression of circ-ITCH was correlated with longer DFS (disease-free survival) (p<0.01) and better OS (overall survival) (p<0.01). Besides, the difference between high expression and low expression of circ-ITCH also included several clinical parameters containing age>60 years, PSA, Gleason score<7, pT3, non-lymph node metastasis, lymph node metastasis and surgical margin-positive. Moreover, Univariate cox's regression analysis was performed to validate the factors that affecting DFS and OS. The results presented the significance of circ-ITCH (high vs low) (HR=0.462 in DFS group and 0.399 in OS group, p<0.01) and other clinicopathological parameters including Gleason score(>7 vs <7) (HR=2.615 in shorter DFS and 3.222 in worse OS, p<0.001), pathologic T stage (pT3 vs. pT2) (HR=2.293 in shorter DFS, p<0.001) and surgical margin status (positive vs. negative) (HR=1.907 in shorter DFS and 2.283 in worse OS, p=0.001), and multivariate cox's regression revealed that circ-ITCH played momentous role in predicting longer DFS and better OS. Taken together, Huang et al. manifested that circ-ITCH exerts as suppressor genes in PC progression through regulating cells activities and signaling pathways in correlation with lower pathological stage and worse lymph node metastasis, which enabled to function as prognostic marker.

Roc curves was also conducted to assess the usage of hsa_circ_0001633, hsa_circ_0001206 and hsa_circ_0009061 as diagnostic marker and the result demonstrated that the area under the curve (AUC) for hsa_circ_0001633, hsa_circ_0001206 and hsa_circ_0009061 was respectively 0.809, 0.774, and 0.711. Furthermore, the clinical features tests for circRNAs indicated that the hsa_circ_0001206 and hsa_circ_0009061 were strongly correlated with Gleason score (G<7 or G>7) (p=0.0018 in hsa_circ_0001206) and pathological stage of patients (PT2, PT3-4) (p=0.045 in hsa_circ_0001206 and p=0.01 in hsa_circ_0009061), suggesting that hsa_circ_0001633, hsa_circ_0001206 and hsa_circ_0009061 had diagnostic and clinical value in PC 47.

## Regulations roles of Circular RNAs in prostate cancer

Attempting to clarify the molecular mechanism of tumorigenesis, multitude of experiments have been established on signaling pathways of process involved in initiation and progression of PC. There are seven imperative signaling cascades prompting the development and progression of PC: Androgen receptor (AR) mediated signaling pathway; NF-kB signaling pathway; Growth factor signaling pathway; Phosphoinositide-3-kinase/AKT signaling pathway; Janus Kinase/signaling transducers and activators of transcription (JAK/STAT) signaling; MAPK pathway; Wnt/β-catenin signaling pathway.

Androgen receptor (AR), a member of the steroid hormone receptor family of ligand-activated nuclear transcription factors, was constitutive of four functional domains responsible for specific ligand binding. The AR signaling pathway played momentous role in normal function of prostate, initiation and maintenance of spermatogenesis 128, 129. Nevertheless, galvanized by stimuli such as androgen production, transforming growth factor, splice variants of full length AR, transcription factors and AR mutants, the aberrant activation of AR signaling pathway contributed to promote the progression of PC and castration-resistant state due to the overexpression and amplification of AR target gene 130. Specially, the clinical effect of inhibiting AR signaling pathway represented a novel aspect of treatment and enzalutamide had been approved by food and drug administration for interrupting the mechanism of AR signaling pathway despite the possibility for developing towards CRPC. Moreover, reactivating the AR signaling pathway also presented irreplaceable role in progression of CRPC and researches focused on the efficacy of therapy in CRPC by targeting AR signaling pathway. To date, accumulating potential targets were disclosed by abundant tests such as YAP signaling and AMPK signaling, which added on the understanding of brand-new mechanism of action in process of PC. To be exactly, YAP, the transcriptional co-activator YES-associated protein, might function as a tumor suppressor in PC and losing of YAP Protein in PC is strongly correlated with Gleason Score increase 130. 5'AMP-activated kinase (AMPK) constituted a hub for cellular metabolic and growth control the essential role of lipogenesis in PC maintenance and progression and pointed out that AMPK-mediated repression of lipogenesis acted compelling function in tumor growth inhibition 132. The discovery of new signal targets enabled the development of much needed new treatment of PC.

The interaction between signaling pathways collectively and invariably regulated the progress of PC. The cross-talk among aforementioned signaling pathways activated or inhibited the downstream signaling molecular and target genes to interfere the tumorigenesis 133. Host of cross-talk capable of mutually influencing the progression of cancer had been demonstrated recent years and AR signaling pathway remained the central and imperative role among all pathways. Several studies had observed the vital role of circRNAs acting as miRNA sponge or indirect modulator for prompting the PC cell proliferation in AR signaling pathway. CircSMARCA5 had been reported to impact the cell cycle and hamper the apoptosis of carcinoma cells after DHT stimulation, which was strongly correlated with AR signaling pathway. Inhibiting circ-SMARCA5 elicited the increased level of cells in G1 phase and decreased level of cells in S phase compared with the control, which indicated that circSMARCA5, an androgen-induced circRNA, exhibited as oncogene in promoting progression of PC. Aforementioned results highlighted the function of circRNA in modulating tumorigenicity via AR signaling pathway, otherwise the potential molecular mechanism between circSMARCA5and AR pathway was required to be further explored 35. CircRNA17 acting as miR-181c-5p reservoir was currently been dissected to be impeded by Enz treatment leading to increase the expression of ARv7. Specially, there were three androgen-response elements (AREs) in the 2kb promoter region in host gene PDLIM5 and ChIP assay found that AR could correlated with the AREs located at 870-884bp upstream of the transcription start site of PDLIM5. Together, Enz suppressed host gene PDLIM5 to decrease the expression of circRNA17 via transcriptional regulation consistent with the traditional AR signaling pathway 134. In addition, Jie et al discovered the upregulation of P-ERK, P-AKT, P-JNK, JNK, β-catenin and GL11 that played crucial role in multiple signaling pathways in overexpression of circ-102004 in PC3 and 22RV1cell lines. Meanwhile, the increased expression of BCL2 and MMP2 was also been found in transfected circ-102004 cell lines resulting in the invasiveness of PC 134. Although a sequence of signaling pathways was potentially associated with the level of circ-102004, the contribution of that in modifying complex signaling networks needed to be further elucidated. Moreover, down-regulating the level of circZNF609 restrained the expression of YAP1, p-AMPK, t-AMPK in PC3 and LNCAP cell lines, which revealed that circZNF609 participated in the YAP and AMPK signaling pathways 39.

## Conclusions and expectations

From the statistics of PC in China and other parts of the world 3, 135, PC remained the most prevalent and vicious cancer threatening human health. The primary diagnostic rate was lower compared with other malignant tumors due to the obsolete specificity and accuracy of blood PSA. However, rapid advances in RNA sequencing and technologies prompted the detection of circRNAs representing a novel aspect of molecular mechanism in tumorigenesis and held some interesting clues about biomarker role in management. N. Vo et al. 136 developed an openly available circRNA compendium, named MiOncoCirc. It could use capture RNA sequencing and include data from clinical cancer samples. For instance, they generated three libraries with exome capture RNA sequencing and detected 1,092 circRNAs from urine samples of PC patients. The results completely overlapped with circRNAs identified in primary prostate adenocarcinoma tissue samples from the MiOncoCirc compendium. It's a reminder that exome capture RNA sequencing may be a promising assay for profiling circRNAs of PC patients in a noninvasive manner. The discoveries of circRNAs went through all the vicissitudes of lives with abundant efforts on attempts and trails and we hoped that not only the existing functions that circRNAs presented but the promising capacities would dedicate to the robust blooming of managements in figuring out conundrums from PC.

**Figure 4:**
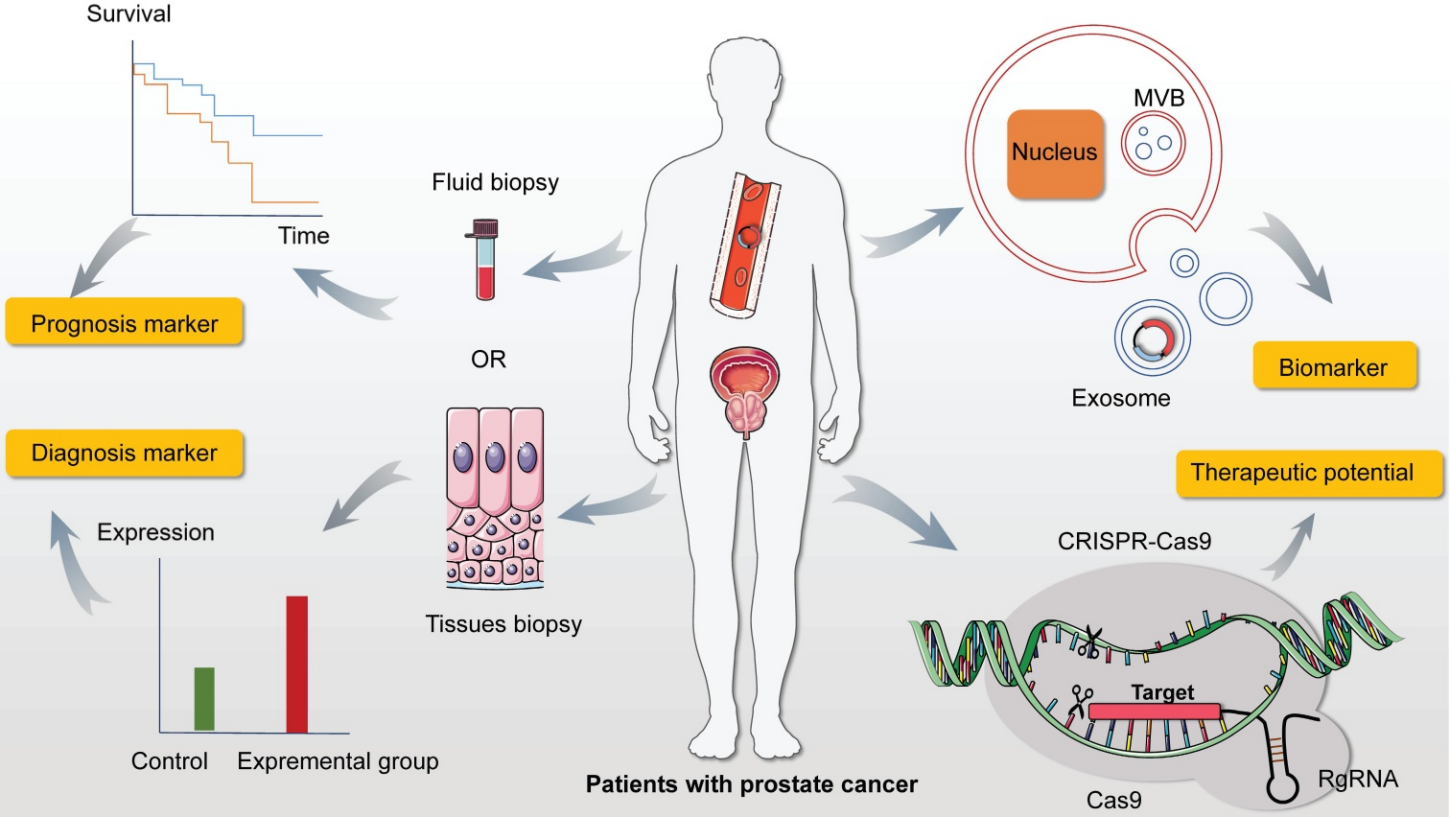
The expectation of circRNAs in prostate cancer. Firstly, abundant and adequate quantities of circRNAs are measured in prostate cancer (PC) from fluid and tissues biopsies with noticeable stability and specificity, which can be conferred the capacities of prognosis and diagnosis. In addition, accumulating researches proved that circRNAs in exosomes exhibit indispensable properties in cancer progression via the function of concentration and targeting. So detecting the level of distinct circRNA in exosomes creates and extends the region of cancer screening and diagnosis in PC. Moreover, it is of importance and well established that CRISPR/Cas9 system directly modify the mutations and mistakes leading to cancer. This novel and practical technology may become a promising candidate with clinical potential in addressing therapeutic issues in PC by suppressing and activating specific molecules.

CircRNAs were characterized by transcriptional regulation for binding miRNA and modulating gene expression. And with its stability and specificity, circRNAs added on a new dimension to primary diagnose and provided new direction for therapeutic measure. To date, the identification and functional research of circRNAs in PC remained limited and elusive. In this review, we summarized the promoting and suppression role in carcinogenic process as well as its additional mechanism in EMT and signaling pathways. We also described the potential efficacy in prognosis, diagnosing and treating. Nevertheless, numerous outstanding issues needed to be confirmed in PC at the area of circRNAs (**Figure 4**).

Recent studies suggested that specific circRNA exonic sequences impact immunity and specifically suggest endogenous m6A modification dampens innate immunity 137. Mice were vaccinated with circFOREIGN, ovalbumin and ovalbumin-expressing B16 melanoma cells successively. The results discovered that mice receiving circFOREIGN have lower tumor growth and have nearly doubled overall survival. Chen et al. 138 discovered that circMALAT1 functioned as a brake in ribosomes to retard Paired box 5 (PAX5) mRNA translation and promote self-renewal of hepatocellular cancer (HCC) stem cells by forming a specific ternary (ribosomecirc-MALAT1-PAX5 mRNA) complex. Recently, Yang et al conducted EpCAM^-/-^ mice via CRISPR/Cas9 technology, which leading to the result that the expression of certain development and glycogen-associated genes was altered in the livers. They elucidated the more integrated mechanism of liver development and carcinogenesis as well as predict the target correlation of the circRNA-miRNA-mRNA network 139. What's more, CRISPR/Cas9 technology was used to be utilized for genome engineering 140. Taken together, could potential targets regulated by circRNAs be processed by CRISPR/Cas9 technology for genome treatment in PC? Could we take advantage of this technology for completing the underling circRNA-miRNA-mRNA network, which might broaden our knowledge of the pathogenesis of PC and eventually improved its treatment strategy.

Exosomes, originated from late endosomes of the endocytic system, are initially discovered by Pan et al. as they research on extracellular cytoplasmic fusion of reticulocyte poly-vesicles 74. The cells communication transferred by mRNA and non-coding RNA including miRNA was mediated by exosomes and that transferred nucleic acids were involved in various life processes. Specially, previous data validated the abundant quantity of circRNAs in exosomes compared to parental cells and more than 1000 exosome circRNAs in serum were represented in discriminating between patients and control 75. Though, Shen et al. 102 elucidated the driving power of circFoxo3 in EMT from exosomes extracted from PC patients, the precise regulatory network of circRNAs in exosomes still needed to be tunneled. Thus, could we discover more typical and stable circRNAs in exosomes exhibiting as cancer-related biomarker? And it is wondering that whether circRNA presents underling oncogenic or suppressive role through targeting exclusive molecules in PC progression?

Other than diverse non-coding RNA, circRNAs covalently were bonded at 3'and 5'ends for forming a successive loop, which was more stable than liner RNAs. The mighty possibility of being novel diagnostic and prognostic marker added on a new dimension to clinical trials in PC. However, the existing discoveries of systematic regulatory network of circRNAs in PC still needed to be further explored and detailed targeting molecules elicited more expansive challenge to scientists. It would be significant to generate promising candidates for targeting therapeutic in PC to release the extensive burden in public health.

## Figures and Tables

**Table 1:** The expression of circRNAs in PC

circRNA	Genome Location	PCcells or tissues	Expression change	Function in PC	References (PMID)
circABCC4	chr13:95813442-95840796	PC-3, DU145, VCaP and LNCaP cells	+	Oncogene	31270953
circHIPK3	chr11:33307958-33309057	22RV1, PC-3, DU145 and LNCaP cells	+	Oncogene	30863152, 31118680
circSMARCA5	chr4:144464662-144465125	22RV1, PC-3, DU145 and LNCaP cells	+	Oncogene	28765045
circZNF609	chr15:64791491-64792365	PC-3 and LNCaP cells	+	Oncogene	31387394
hsa_circ_0001206	chr22:21288066-21288532	PC-3, DU145, and LNCaPcells	-	Suppressor gene and biomarker	31198063, 32919302
hsa_circ_0001633	chr6:107824860-107827631	PC tissues	-	Suppressor gene	31198063
hsa_circ_0009061	chr1:23356961-23377013	PC tissues	-	Suppressor gene	31198063
circAMOTL1L	chr22:35948707-35948901	PC-3 and DU145 cells	-	Suppressor gene	30531834
circ102004	chr17	PC-3 and 22RV1 cells	+	Oncogene	30219508
circMYLK	chr3:123332641-123332832	PC-3, DU145, LNCaP and PC-3MIE8 cells	-	Suppressor gene	29798970
circRNA17	chr4:95561426-95578698	EnzR-C4-2 cells	+	Oncogene	30674872
circFoxo3	chr6:108984657-108986092	PC-3, DU145, VCaP and LNCaP cells	-	Suppressor gene	31593800
circ0005276	chrX:123022468-123026623	PC-3, DU145, VCaP and LNCaP cells	+	Oncogene	31624242
hsa_circ_0044516	chr17:48263677-48271402	PC-3, 2B4 and 22RV1 cells	+	Oncogene and biomarker	31625175
circ-UCK2	chr1:165859440-165877108	EnzR-C4-2 cells	-	Suppressor gene	31844675
circ_KATNAL1	chr13:30801548-30857928	22Rv1, DU145, LNCaP and PC-3 cells	-	Suppressor gene	31800303
hsa_circ_0001165	chr20:46252654-46262380	PC-3M IE8 cells	+	Oncogene	31882179
hsa_circ_0001085	chr2:191765289-191789319	PC-3M IE8 cells	+	Oncogene	31882179
hsa_circ_0004916	chr2:44428324-44436466	PC-3M IE8 cells	+	Oncogene	31882179
circFMN2	chr1:240458121-240497529	PC-3, LNCaP, VCaP and DU145 cells	+	Oncogene	32526477
circ0016068	chr1:203274663-203278729	PC-3, DU145, VCaP and 22RV1 cells	+	Oncogene	32984325
circMBOAT2	chr2:9083315-9098771	PC-3, LNCaP, VCaP, DU145 and C4-2B cells	+	Oncogene	32645691
circLMTK2	chr7:98190727-98194572	PC-3 and LNCaP cells	-	Suppressor gene	31760099

**Table 2:** CircRNAs function as microRNA sponges in PC

circRNA	microRNA sponged	PC cells	References (PMID)
circABCC4	miR-1182	PC-3, DU145 cells	31270953
circHIPK3	miR-338-3p	PC-3, DU145 cells	31118688
circHIPK3	miR-193a-3p	PC-3, DU145 cells	30863152
circZNF609	miR-186-5p	PC-3, LNCAP cells	31387394
hsa_circ_0001206	miR-1285-5p	DU145 cells	31198063
circAMOTL1L	miR-193a-5p	PC-3 cells	30531834
circMYLK	miR-29a	PC-3, PC-3M IE8 cells	29798970
hsa_circ_0044516	miR-29a-3p	PC-3,2B4, RV221cells	31625175
circ_KATNAL1	miR-145-3p	22Rv1, DU145, LNCaP and PC-3 cells	31800303
circFMN2	miR-1238	PC-3, LNCaP, VCaP and DU145 cells	32526477
circ0016068	miR-330-3p	PC-3, DU145, VCaP and 22RV1 cells	32984325
circMBOAT2	miR-1271-5p	PC-3, LNCaP, VCaP, DU145 and C4-2B cells	32645691
circLMTK2	miR-183	PC-3 and LNCaP cells	31760099

**Table 3:** The targets of circRNAs in PC cell lines

circRNA	Target	PC cells	References (PMID)
circABCC4	FOXP4	PC-3, DU145 cells	31270953
circHIPK3	MCL1, ADAM17	PC-3, DU145 cells	31118688, 30863152
circZNF609	MMP-9, vimentin, YAP1, AMPK	PC-3, LNCAP cells	31387394
hsa_circ_0001206	Smad4	DU145 cells	31198063
circAMOTL1L	Pcdha8, E-cadherin, vimentin, β-catenin	PC-3 cells	30531834
circRNA17	ARv7	C4-2, CWR22Rv1 cells	30674872
circ_KATNAL1	WISP1, MMP-2, MMP-9, Caspase-3, Caspase-8, Caspase-9, PARP	22Rv1, DU145, LNCaP and PC-3 cells	31800303
circFMN2	LHX2	PC-3 cells	32526477

## Abbreviations

AR: androgen receptor
ADT: androgen deprivation therapy
Amotl1: angiomotin-like 1 gene
AUC: area under the curve
AREs: androgen-response elements
AMPK: 5'AMP-activated kinase
BPH: benign prostatic hyperplasia
PSA: blood prostate specific antigen
CircRNAs: circular RNAs
CRPC: castration resistant prostate cancer
ciRNA: circular intronic RNA
DOX: Docetaxel
DFS: disease-free survival
ecircRNA: exoniccircRNA
EIciRNA: exon-intron RNA
EMT: epithelial-mesenchymal transition
EVs: extracellular vehicles
EnzRPCa: enzalutamide resistant prostate cancer
FUS: fused in sarcoma
FDA: food and drug administration
icircRNA: intregenic RNA
JAK/STAT: Janus Kinase/signal transducers and activators of transcription
IncRNAs: long non-coding RNAs
miRNA: microRNA
MREs: microRNA response elements
MCL1: myeloid cell leukemia 1
OS: overall survival
PC: prostate cancer
PIN: prostatic intraepithelial neoplasia
QKI: Quaking
RBPs: RNA binding-proteins
XIAP: X-linked inhibitor of apoptosis protein
